# Severe Nocturnal and Postexercise Hypoxia in Children and Adolescents with Sickle Cell Disease

**DOI:** 10.1371/journal.pone.0097462

**Published:** 2014-05-30

**Authors:** Isabelle Halphen, Caroline Elie, Valentine Brousse, Muriel Le Bourgeois, Slimane Allali, Damien Bonnet, Mariane de Montalembert

**Affiliations:** 1 Pediatric Emergency Department, Hospital Necker, APHP, Paris, France; 2 Paris Descartes University, Paris, France; 3 Department of Biostatistics, Hospital Necker, APHP, Paris, France; 4 Pediatrics Department and Sickle Cell Clinic, Hospital Necker, AP-HP, Paris, France; 5 Pediatric Pneumology and Allergology Department, Hospital Necker, APHP, Paris, France; 6 Pediatric Cardiology Department, M3C–Necker, AP-HP, Paris, Paris Descartes University, France; Instituto de Higiene e Medicina Tropical, Portugal

## Abstract

Hypoxia is a common feature in children with sickle cell disease (SCD) that is inconsistently associated with painful crises and acute chest syndrome. To assess the prevalence and risk factors of hypoxia, we recorded daytime, nocturnal, and postexercise pulse oximetry (SpO_2_) values in 39 SCD patients with a median age of 10.8 years. Median daytime SpO_2_ was 97% (range, 89%–100%), and 36% of patients had daytime hypoxia defined as SpO_2_<96%. Median nocturnal SpO_2_ was 94.7% (range, 87.7%–99.5%), 50% of patients had nocturnal hypoxia defined as SpO_2_≤93%, and 11(37%) patients spent more than 10% of their total sleep time with SpO_2_<90%. Median postexercise SpO_2_ was 94% (range, 72%–100%) and 44.7% of patients had postexercise hypoxia defined as an SpO_2_ decrease ≥3% after a 6-minute walk test. Among patients with normal daytime SpO_2_, 35% had nocturnal and 42% postexercise hypoxia. Compared to 9 patients without daytime, nocturnal, or postexercise hypoxia, 25 patients with hypoxia under at least one of these three conditions had greater anemia severity (*P* = 0.01), lower HbF levels (*P* = 0.04), and higher aspartate aminotransferase levels (*P* = 0.03). Males predominated among patients with postexercise hypoxia (*P* = 0.004). Hypoxia correlated neither with painful crises nor with acute chest syndrome. Of 32 evaluable patients, 6 (18.8%) had a tricuspid regurgitation velocity ≥2.6 m/s, and this feature was associated with anemia (*P* = 0.044). Median percentage of the predicted distance covered during a 6-minute walk test was 86% [46–120]; the distance was negatively associated with LDH (*P* = 0.044) and with a past history of acute chest syndrome (*P* = 0.009). In conclusion, severe episodes of nocturnal and postexercise hypoxia are common in children with SCD, even those with normal daytime SpO_2_.

## Introduction

Sickle cell disease (SCD) is associated with chronic hemolysis, resistance to nitric oxide (NO) bioactivity, small-vessel obstruction, and ischemia-reperfusion injury. Respiratory complications such as acute chest syndrome (ACS) and pulmonary hypertension (PH) are the most common identifiable causes of premature death in adults with SCD [1]. Many studies of children with SCD showed hypoxia during the day, while sleeping, or after exercising [2]–[11]. The consequences of hypoxia in SCD children are unclear. In some studies, hypoxia was associated with pain [7] and ACS [11], but other studies failed to replicate these findings [3], [8]. Associations with neurological complications have been reported [9], [10]. While hypoxia in children with SCD was associated with elevation of the tricuspid regurgitation velocity (TRV) [4], [5], [12], there is still intense controversy over the indiscriminate use of the TRV to estimate pulmonary pressure in this condition. TRV appears to have limitations in specificity, but may indicate the presence of PH, which must be confirmed by right heart catheterization [13], [14]. None of the available studies in children with SCD simultaneously evaluated daytime, nocturnal, and postexercise oxygen saturations, together with tonsil size, painful events, lung function, echocardiographic data including TRV, blood markers for hemolysis, and performance on the 6-minute walk test (6 MWT). The 6 MWT has shown good reliability and is increasingly used in children affected with chronic disease to evaluate their sub-maximal functional exercise capacity [15].

Here, our primary objective was to assess the prevalence of hypoxia in children with SCD by performing, not only the usual daytime measurements, but also measurements during three nights and after a 6 MWT. Secondary objectives were to identify risk factors for daytime, nocturnal, and postexercise hypoxia and to identify correlations linking hypoxia to vasoocclusive crises (VOCs), ACS, and TRV.

## Methods

### Study Design

We conducted a prospective single-center study in consecutive children with SCD who had either the homozygous SS or the S/β_0_ thalassemia genotype and who were seen at the SCD clinic of the Necker Hospital, Paris, France. Our local institutional review board (*Conseil Ethique Necker-Enfants Malades*) approved the study. Written informed consent was obtained for all patients from the parents/guardians, and from the minors who were old enough to understand. The data used in this study were anonymized.

The SCD phenotype was confirmed by hemoglobin (Hb) electrophoresis and/or high-performance liquid chromatography. Patients who were in a chronic transfusion program were excluded. All investigations were performed in patients in stable condition, defined as a 3-month period without admission for painful events and without blood transfusion therapy.

We included 42 consecutive patients. Three patients were excluded because their echocardiograms showed asymptomatic congenital heart disease (pulmonary valve stenosis in 2 and persistent arterial duct in 1). Among them, 38 had the SS genotype and one had S/β_0_ thalassemia.

### Clinical and Laboratory Data

VOCs were defined as painful episodes having no other explanation than vasoocclusion and requiring therapy prescribed by a healthcare professional in a medical setting such as a hospital, clinic, or emergency room. The number of VOCs during the year before inclusion was determined retrospectively by hospital chart review. The number of VOCs during the year after inclusion was to be determined prospectively by having the patients and/or parents complete a standardized form; however, few patients/parents completed the forms regularly and we therefore used the number of VOCs recorded in the hospital charts. We also reviewed the hospital charts for data on the history of ACS (defined as a new pulmonary infiltrate combined with one or more of the following: fever, cough, sputum production, tachypnea, dyspnea, and new-onset hypoxia). Hydroxycarbamide treatment was recorded. Tonsil size and blood pressure were measured prospectively. The day when the lung function tests were performed, we measured Hb; reticulocyte, leukocyte, and platelet counts; and the fetal Hb level (HbF, determined using the HPLC system from Bio-Rad). As markers for hemolysis, we recorded the serum levels of aspartate aminotransferase (AST), total bilirubin, and lactate dehydrogenase (LDH), determined using standard methods.

Oxygen saturation (SpO_2_) was measured by finger pulse oximetry at rest using a MasimoRadicalSET pulse oximeter (Masimo, Irvine, CA). We defined daytime hypoxia as SpO_2_<96% [11]. Nocturnal finger pulse oximetry was recorded using a Nonin device (Nonin Medical, Plymouth, MN) during three consecutive nights, at home, in 30 patients. We considered the median nighttime SpO_2_ over the three nights and the worst mean SpO_2_ during any of the three nights. We defined nocturnal hypoxia as SpO_2_≤93% [16]. We also recorded the percentage of sleep time spent with SpO_2_<90%.

An unencouraged 6 MWT was conducted as recommended by the American Thoracic Society Pulmonary Function Standards Committee [17]. The 6 MWT was performed on a 20-m straight track. Patients were instructed to cover the largest possible distance in 6 minutes as follows: “the objective of this test is to walk as quickly as you can during 6 minutes. You may slow down or stop if you need to, and restart the walk as soon as you can. I will announce every minute that goes by. Your goal is to walk as fast as you can for 6 minutes. I will write down the distance you walked.” Turns were made on both ends of the 20-m track. The distance was recorded with a lap counter. At the end of the test, patients were asked to stand still, and the distance covered in the final partial lap was measured. The total distance covered was calculated by multiplying the number of laps (back and forth once) by 40 m then adding the final lap. The total distance walked was rounded off to the nearest meter. We expressed the 6 MWT distances as percentages of the predicted distance for age, using the reference values reported by Geiger et al. in 2007 [18]. We defined postexercise hypoxia as an SpO_2_ decline ≥3% versus baseline [5]. The postexercise SpO_2_ recording failed in 1 patient.

### Echocardiography

Echocardiographic data were acquired from standard parasternal views and from apical two- and four-chamber views (Vivid-7, General Electric Vingmed, Horten, Norway). When children were over 5 years old and able to cooperate, standard M-mode and 2D images were obtained at end-expiratory apnea and stored in cine-loop format from three consecutive beats. M-mode measurements were performed according to recommendations of the American Society of Echocardiography. Right atrial pressure (RAP) was defined as normal (5 mmHg) if respiratory changes in inferior vena cava diameter were >40%. Right ventricular systolic pressure was computed as 4 · TRV^2^+ RAP. Mean pulmonary artery pressure was computed as 4 · protodiastolic velocity of pulmonary regurgitation^2^ +7 mmHg. Diastolic pulmonary artery pressure was computed as 4 · telediastolic velocity of pulmonary regurgitation^2^ +7 mmHg.

### Lung Function Tests

Spirometry, plethysmography, and measurement of lung diffusion capacity for carbon monoxide (DLCO) were performed in all children as recommended by the American Thoracic Society/European Respiratory Society. DLCO was adjusted for Hb concentration. We recorded forced expiratory volume in 1 second (FEV), forced vital capacity (FVC), and FEV/FVC; and we classified the ventilation phenotypes as normal, restrictive, obstructive, or mixed according to guidelines issued by the American Thoracic Society/European Respiratory Society [19].

### Data Analysis

For between-group comparisons, we used Fisher’s exact test for categorical variables and Wilcoxon test’s for continuous variables. Relations between quantitative variables were assessed using Spearman’s correlation coefficient. *P* values<0.05 were considered significant. Statistical analysis was performed with R software (http://cran-project.org).

## Results

### Clinical and Laboratory Features

Table 1 and Table 2 list the main features of the 39 study patients. None had clinical symptoms suggesting obstructive sleep apnea.

**Table 1:** Main characteristics of the 39 children and adolescents with sickle cell disease (part 1).

	N	%
Male gender	14	36
Enlarged tonsils	16/37	43
HC treatment	9	23
History of ACS	15	38.5

**Table 2:** Main characteristics of the 39 children and adolescents with sickle cell disease (part 2).

	Median	Range	Normal range
Age (years)	10.8	5.7–17	
BMI (Kg/m^2^)	17	13–24	
VOC in the past year	0	0–7	
VOC in the next year	0	0–6	
Basal heart rate (bpm)	97	75–122	
Systolic blood pressure (mmHg)	108	87–132	
Diastolic blood pressure (mmHg)	65	47–85	
Hemoglobin (g/dL)	7.9	5.2–10.6	
Leukocytes (Giga/L)	10.8	5.7–21.5	
Reticulocytes (Giga/L)	239	43–443	
Fetal hemoglobin (%)	9.2	0.8–28	
Platelets (Giga/L)	379	118–742	
Aspartate aminotransferase (IU/L)	62	35–132	9–40
Alanine aminotransferase (IU/L)	24	11–68	7–40
Total bilirubin (µmol/L)	42	13–163	0–17
Lactate deshydrogenase (IU/L)	1421	618–1893	125–243
Creatinine (µmol/L)	36	20–57	20–75
6 MWT distance (% predicted distance)	86	46–120	

HC, hydroxycarbamide; ACS, acute chest syndrome; BMI, body mass index; VOC, vasoocclusive crisis.

### Lung and Heart Function

Lung function tests were abnormal in 15 (38.5%) patients, of whom 4 had an obstructive pattern, 8 a restrictive pattern, and 3 a mixed pattern. Hb-adjusted DLCO was normal in all but 2 patients, whose values were 69% and 72% of predicted, respectively.

Of the 38 patients who underwent echocardiography, 7 had mild left ventricular dilation (Z score>2). All patients had normal left ventricular ejection fraction values (>60%). TRV was measurable in 32/38 patients and was ≥2.6 m/s in 6 (19%). Protodiastolic pulmonary regurgitation velocity (estimated mean pulmonary artery pressure) was measurable in 19/38 patients, none of whom had values greater than 2.0 m/s. Of 16/38 patients for whom telediastolic pulmonary regurgitation velocity (estimated diastolic pulmonary artery pressure) was measurable, none had values greater than 1.4 m/s. Overall, systolic, mean, and/or diastolic pulmonary artery pressures were assessable in 35/38 patients, none of whom had elevated values.

### Prevalence of Hypoxia

#### Day

Of the 39 patients, 14 (36%) had SpO_2_ values lower than 96% during the day. Median daytime SpO_2_ was 97% (range, 89%–100%).

#### Night

Of the 30 patients with nocturnal SpO_2_ recordings, 15 (50%) had SpO_2_ values ≤93%. The median of the lowest SpO_2_ values was 93.3% (range, 87.4%–99.5%). The median nighttime oxygen saturation was 94.7% (range, 87.7–99.5). Of the 30 patients, 11 (37%) spent more than 10% of their total sleep time with SpO_2_<90%.

#### Postexercise

Of the 38 patients with post-6 MWT data, 25 (65%) had SpO_2_<96% and 10 (26%) had SpO_2_<90%. Median postexercise SpO_2_ was 94% (range, 72%–100%). Compared to baseline, the SpO_2_ decline after the 6 MWT was ≥3% in 17 (44.7%) patients.

### Associations across Daytime, Nocturnal, and Postexercise Spo_2_ Values

Interpretable SpO_2_ recordings were available in all three conditions for 34 patients.

A significant association was found between daytime and nocturnal SpO_2_ values (*P* = 0.02). Among children with normal daytime SpO_2_ values, 35% had nocturnal hypoxia (compared to 80% of those with daytime hypoxia). Daytime and postexercise SpO_2_ values were not significantly associated with each other (*P* = 0.62). The proportion of children with a postexercise SpO_2_ decline ≥3% was 42% in the group with normal daytime SpO_2_ and 50% in the group with daytime hypoxia. We found no significant association between nocturnal and postexercise SpO_2_ (*P* = 0.10).

### Factors Associated with Hypoxia

Hypoxia was found under at least one of the three measurement conditions in 25 (73.5%) patients and under none of the three conditions in 9 patients. Table 3 compares these two groups. Hypoxia was associated with greater anemia severity (*P* = 0.01), lower HbF (*P* = 0.04), and higher AST (*P* = 0.03). Hypoxia was associated neither with a past history of ACS nor with the number of VOCs. Hypoxia was less common in hydroxycarbamide-treated patients, but the difference was not statistically significant (*P* = 0.31).

**Table 3:** Risk factors for at least one type of hypoxemia (day, night, and postexercise).

	No hypoxemia (n = 9)	Hypoxemia (n = 25)	*P* value
**Medical history**			
Age (yrs)	9.0 (5.7–17.0)	10.5 (6.2–16.8)	0.56
Male gender	1 (11%)	11 (44%)	0.11
BMI (Kg/m^2^)	16.3 (13.3–18.8)	16.1 (13.9–22.2)	0.33
Enlarged tonsils	4 (50%)	12 (48%)	1
N of VOCs in past year	0 (0–7)	0 (0–2)	0.14
Hydroxycarbamide treatment	3 (33%)	3 (12%)	0.31
History of at least one ACS episode	3 (33%)	10 (40%)	1
Abnormal lung function test	2 (22%)	11 (44%)	0.43
**Laboratory tests**			
**Hemoglobin (g/dL)**	**8.5 (7.5–10.2)**	**7.5 (5.2–10.6)**	**0.01**
Leukocytes (Giga/L)	9.4 (5.8–16.0)	11.1 (5.7–21.5)	0.30
Reticulocyte count (Giga/L)	220 (43–276)	246 (103–443)	0.14
Lactate dehydrogenase (IU/L)	1126 (901–1606)	1467 (849–1893)	0.15
Total bilirubin (µmol/L)	41 (17–130)	48 (22–163)	0.60
**Aspartate aminotransferase (IU/L)**	**49 (46–100)**	**63 (39–132)**	**0.03**
**Fetal hemoglobin (%)**	**11.4 (2.6–28)**	**5.7 (0.8–20.2)**	**0.04**
Creatinine (µmol/L)	37 (31–40)	36 (20–57)	0.40
6 MWT distance (% predicted distance)	86 (46–120)	86 (49–119)	1

BMI, body mass index; VOC, vasoocclusive crisis; ACS, acute chest syndrome; 6 MWT, 6-minute walking test.

Lower values for the lowest nocturnal SpO_2_ (Table 4) were associated with greater anemia severity (*P* = 0.006), lower HbF levels (*P* = 0.01), abnormal lung function tests (*P* = 0.003), daytime SpO_2_ (*P* = 0.03), and postexercise SpO_2_ (*P* = 0.04) but not with larger tonsil size, number of VOCs, or history of ACS. The worst nocturnal SpO_2_ value was strongly associated with the median nocturnal SpO_2_ (*P* = 10^−5^) and with the percentage of sleep time spent with SpO_2_<90% (*P* = 0.0007).

**Table 4:** Clinical and laboratory characteristics of patients according to lowest nocturnal SpO_2._

	>93% (n = 15)	≤93% (n = 15)	*P* value
**Medical history**			
Age (yrs)	10.1 (5.7–17.0)	9.1 (6.3–16.8)	0.65
Male gender	3 (20%)	7 (46.7%)	0.12
BMI (Kg/m^2^)	16.3 (13.3–19.6)	15.7 (13.9–21.4)	0.60
Enlarged tonsils	6 (42.9%)	7 (46.7%)	0.84
N of VOC in the past year	0 (0–7)	0 (0–2)	0.75
Hydroxycarbamide treatment	4 (26.7%)	2 (13.3%)	0.65
History of at least one ACS episode	5 (33.3%)	6 (40%)	0.70
**Abnormal lung function test**	**2 (13.3%)**	**10 (66.7%)**	**0.003**
**SpO_2_ values**			
**Daytime SpO_2_**	**98 (89–100)**	**95 (92–99)**	**0.03**
**Postexercise SpO_2_**	**97 (79–100)**	**92 (72–100)**	**0.04**
**>10% of sleep time with SpO_2_<90%**	**1 (6.7%)**	**10 (66.7%)**	**0.0007**
**Laboratory tests**			
**Hemoglobin (g/dL)**	**8.4 (6.9–10.6)**	**7.5 (5.2–9.5)**	**0.006**
Leukocytes(Giga/L)	9.4 (5.8–16)	10.8 (5.7–21.5)	0.19
Reticulocyte count (Giga/L)	220 (43–276)	246 (121–443)	0.07
Lactate dehydrogenase (IU/L)	1196 (901–1683)	1456 (849–1893)	0.66
Total bilirubin (µmol/L)	36(17–130)	55 (22–163)	0.12
Aspartate aminotransferase (IU/L)	51 (39–132)	63 (48–93)	0.10
**Fetal hemoglobin (%)**	**12.4 (2.6–28)**	**5.4 (0.8–11.7)**	**0.01**
Creatinine (µmol/L)	36 (20–43)	36 (22–51)	0.91
6 MWT distance (% predicted distance)	86 (46–120)	87 (50–119)	0.66

BMI, body mass index; VOC, vasoocclusive crisis; ACS, acute chest syndrome; 6 MWT, 6-minute walking test.

We compared the 17 patients with and the 21 patients without a postexercise SpO_2_ decline ≥3% (Table 5). Factors significantly associated with a decline ≥3% were male gender (*P* = 0.004) and a higher percentage of sleep time spent with SpO_2_<90% (*P* = 0.048).

**Table 5:** Clinical and laboratory characteristics according to the SpO_2_ decline induced by the 6-minute walking test.

	Decline<3%(n = 21)	Decline≥3% (n = 17)	*P* value
**Medical history**			
Age (yrs)	10.5 (5.7–17.0)	11.5 (6.2–15.6)	0.32
**Male gender**	**3 (14.3%)**	**10 (58.8%)**	**0.004**
BMI (Kg/m^2^)	16.1 (13.3–24.0)	16.9 (14.0–21.4)	0.62
Enlarged tonsils	8 (40%)	7 (43.8%)	0.82
N of VOC in the past year	0 (0–7)	0 (0–6)	0.22
Hydroxycarbamide treatment	6 (28.6%)	3 (17.6%)	0.48
Past history of at least one ACS episode	8 (38.1%)	7 (41.2%)	0.85
Abnormal lung function test	6 (28.6%)	8 (47.1%)	0.24
**SpO_2_ values**			
Daytime SpO_2_	98 (89–100)	96 (92–100)	0.44
Lowest nocturnal SpO_2_	95.5 (87.5–99.5)	92.0 (87.8–96)	0.051
**% sleep time with SpO_2_<90%**	**0.3 (0–90.6)**	**9 (0.1–68.2)**	**0.048**
**Laboratory tests**			
Hemoglobin (g/dL)	8.2 (6.6–10.2)	7.5 (5.2–10.6)	0.08
Leukocytes (Giga/L)	10.7 (5.7–16.0)	11.4 (7.7–21.5)	0.92
Reticulocyte count (Giga/L)	232 (43–443)	239 (104–357)	0.90
Lactate dehydrogenase (IU/L)	1130 (618–1731)	1467 (849–1893)	0.17
Total bilirubin (µmol/L)	47 (13–130)	39 (13–161)	0.55
Aspartate aminotransferase (IU/L)	56 (35–132)	66 (39–93)	0.06
Fetal hemoglobin (%)	10.1 (2.6–28)	7.1 (3.9–20.2)	0.46
Creatinine (µmol/L)	37 (20–53)	35 (22–57)	0.20
6 MWT distance (% predicted distance)	92 (46–120)	86 (73–102)	0.71

BMI, body mass index; VOC, vasoocclusive crisis; ACS, acute chest syndrome; 6 MWT, 6-minute walking test.

### Factors Associated with Elevated Tricuspid Regurgitation Velocity (TRV)

TRV elevation to ≥2.60 m/sec was found in six (18.8%) of 32 children in whom it could be measured. Importantly, the 4 patients with TRV>2.6 m/s had normal estimated mean and/or diastolic pulmonary artery pressures.

TRV≥2.60 m/sec was associated with greater anemia severity (8.2 g/dL [range, 6.5–10.6] versus 7.4 [range, 6.4–8.1], *P* = 0.045). In contrast, TRV≥2.60 m/sec was not associated with any of the other study parameters, including hypoxia under any of the three measurement conditions (data not shown).

### 6-Minute Walking Test (6 MWT)

**Figure 1:**
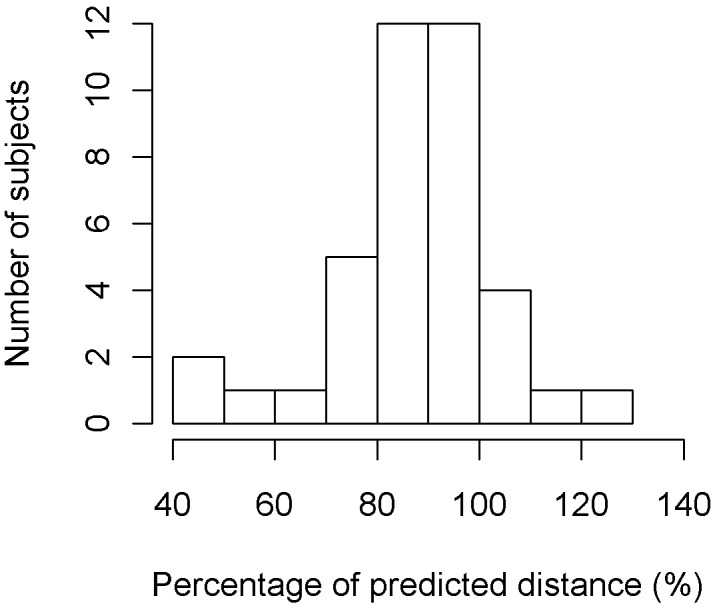
Distribution of the patients according to distance walked during the 6-minute walking test, expressed as % of the expected value.

Median distance walked during the 6 MWT was 547 m (range, 303–702 m). The median percentage of predicted distance was 86% [46–120] (Figure 1). The 6 MWT distance correlated negatively with the LDH levels (*P* = 0.044, rho = –0.35). It was also strongly associated with a past history of ACS: median, 94% [50–120] in children with no past history of ACS and 83% [46–95] in those with at least one ACS episode (*P* = 0.009].

## Discussion

We used pulse oximetry to assess the prevalence of hypoxia in children and adolescents with SCD during the day, at night during sleep, and after exercise. We found that 36% of patients had hypoxia under steady-state daytime conditions, 50% at night while sleeping, and 45% after a 6 MWT. These results were observed in children who did not have the most severe forms of the disease as, in our unit, such children receive chronic monthly blood transfusions, which was an exclusion criterion for the present study.

Many studies used pulse oximetry to assess hypoxia in children with SCD [2]–[11] but, to our knowledge, none obtained measurements during the day, during sleep, and after exercise in the same patients. Pulse oximetry has been shown to reliably assess oxygen saturation in patients with SCD [20], [21]. Measurement variability across three measurements obtained in the same children over a 12-month period was noted when the initial SpO_2_ value was ≤92% [22]. Whether this intraindividual variability was related to modifications in the health condition of the patient, modifications in Hb levels, or proximity of the SpO_2_ value to the inflection point of the oxygen dissociation curve remained unclear.

In keeping with earlier data [3]–[6], factors associated with hypoxia under at least one of our three measurement conditions were greater anemia severity, lower HbF, and higher AST values (indicating greater hemolytic activity). VOC and ACS were not different between the groups with and without hypoxia under at least one of the three conditions. Importantly, normal daytime SpO_2_ did not predict absence of nocturnal or postexercise hypoxia, although significant associations were found between these variables. Thus, among the patients with normal daytime SpO_2,_ one-third had nocturnal hypoxia and 42% a postexercise SpO_2_ decline ≥3%. Nocturnal hypoxia was frequently severe: 37% of our unselected patients in stable condition spent more than 10% of their sleep time with SpO_2_<90%. Nocturnal hypoxia was associated with greater anemia severity, low HbF, daytime SpO_2_, postexercise SpO_2_ decline, and abnormal lung function tests but not with tonsil size. In a study of 95 children (78% with the SS genotype) having a mean age of 8.2 years (range, 2.1–16.9), the percentage of sleep time spent with SpO_2_<90% was 11.1% (range, 0–99.6), and low nocturnal SpO_2_ was significantly associated with a greater number of VOCs [7]. The absence of a significant association in our study between nocturnal SpO_2_ and number of VOCs may be ascribable to our small sample size and/or to the fact that we recorded only VOCs requiring admission.

Nocturnal hypoxia was associated with neurological complications of SCD in earlier studies [9], [10]. We did not record transcranial Doppler velocities. None of the patients experienced overt stroke before or after study enrollment.

The 6-MWT, i.e., an exercise of moderate intensity, induced a ≥3% decline in SpO_2_ in 45% of patients, and 26% of the overall population had postexercise SpO_2_ values <90%. Similarly, in another study, 34% of children with SCD had postexercise hypoxia [23]. Postexercise hypoxia was significantly more common in our studies in males than in females. In an earlier study, steady-state SpO_2_ was lower in males than in females [3]. Greater NO bioavailability and NO responsiveness has been reported in females than in males with SCD [24]. In keeping with this finding, in a study of 16 654 SCD-related deaths between 1979 and 2005 in the US, women had an older mean age at death compared to men (36.9 years [95% confidence interval, 36.5–37.4] vs. 33.4 years [33.0–33.7]) [25].

Anemia only partly explains the hypoxia seen in SCD. According to one study, anemia may explain only 5% of the arterial oxygen desaturation in children with SCD [3]. One factor that may contribute to decrease oxygen saturation in SCD is diminished affinity of the sickle Hb for oxygen related to an increased content of erythrocyte 2,3-biphosphoglycerate [26]. Chronic hemolysis may also contribute to hypoxia. Significant associations have been reported in SS children between hypoxia at rest and hemolysis, and between postexercise hypoxemia and hemorrheological abnormalities [23]. Also, reduced NO availability related to hemolysis may result in pulmonary vasculopathy responsible for ventilation-perfusion mismatching and limited oxygen uptake by Hb [27]. A correlation has been found between hypoxemia severity and markers for cellular activation (soluble L-selectin, P-selectin, VCAM-1, and leukotriene B_4_), suggesting a pathophysiological explanation for the onset of complications in hypoxemic SCD patients [6]. In addition, vascular endothelial lesions may be exacerbated by reoxygenation phases, which are known to trigger inflammation [28]. This last possibility suggests a need for carefully appraising the risk/benefit ratio of sports participation in patients with SCD. Although improving cardio-pulmonary fitness is of crucial importance, additional data on potential risks related to postexercise hypoxia are needed.

Of 32 patients with TRV measurements in our study, 6 (19%) had TRV values ≥2.6 m/s, in keeping with reports that the prevalence of elevated TRV ranged from 11% to 30% among children with SCD [4], [29]–[40]. TRV≥2.6 m/s was associated with anemia but not with hemolysis; this last finding is probably ascribable to our small sample size. Of note, none of our patients had pulmonary regurgitation velocities suggesting elevated pulmonary vascular resistance. We are aware that TRV has low sensitivity as a PH screening tool in adults. Our patients did not undergo right heart catheterization and, therefore, we cannot rule out that some of them had undiagnosed PH. Of note, the patients with the highest TRV values in our study had LV dilatation. Confirming the relation between elevated TRV and LV filling pressures would require pulmonary wedge pressure or diastolic transpulmonary gradient measurement, now suggested as the reference standard tool for diagnosing postcapillary PH.

TRV elevation was associated with a decrease in the 6 MWT distance in several studies of children with SCD [4], [32]. In contrast, we found no association between TRV and the 6 MWT distance. The limited distances achieved by our patients during the 6 MWT were comparable to those recently reported in children with SCD [40], [41], and in children with different chronic conditions such as juvenile idiopathic arthritis, hemophilia, spina bifida, and mildly to moderately symptomatic cystic fibrosis [42], [43]. Interestingly, Waltz et al showed that a high level of anemia, low fetal hemoglobin expression, and low red blood cell deformability independently predicted poor 6 MWT performance [41]. We confirm the association between reduced 6 MWT distances and severe hemolysis as indicated by increased LDH levels in children with SCD; as well as the negative impact of prior ACS, which to our knowledge had not been reported previously. Of note, a recent study found that a decrease in the 6 MWT distance correlated with silent infarcts in children with SCD, suggesting a role for underlying chronic hypoxia [40].

The best strategies for minimizing hypoxia-related damage are unclear. In 3 children with SCD, the introduction of hydroxycarbamide therapy was followed by the resolution of chronic hypoxia, and this effect was not entirely ascribable to increases in Hb and HbF levels [44]. The respective indications of hydroxycarbamide, chronic blood transfusion, and hematopoietic stem cell transplantation in children with SCD and hypoxia and/or TRV elevation are not established [45], [46]. Nocturnal oxygen therapy may decrease the risk of developing hypoxemia-related vasculopathy. In a phase I controlled trial, overnight auto-adjusting positive airway pressure, with supplemental oxygen when nocturnal oxygen saturation was below 94%, improved sleep-related breathing disorders and one aspect of cognition in children with SCD, without inducing bone marrow suppression [47].

In conclusion, the prevalence of severe episodes of oxygen desaturation is high among children and adolescents with SCD. Normal daytime SpO_2_ values do not rule out nocturnal and/or postexercise desaturation episodes. Hypoxia is related to anemia, low HbF, and hemolysis. Many patients have a limited 6 MWT distance. The long-term consequences of hypoxia are unclear, especially regarding the risk of developing PH. Large-scale, prospective, controlled trials are needed and should include investigations of the effects of hydroxycarbamide and oxygen therapy.
